# Supplementary material for: Chromothripsis during telomere crisis is independent of NHEJ, and consistent with a replicative origin
Source: Genome Res. 2019 May;29(5):737–49. doi: 10.1101/gr.240705.118 (PMC6499312; doi:10.1101/gr.240705.118)
Supplement: Supplemental Material [file supp_gr.240705.118_Supplemental_file_1.zip › contigs/annotated_contigs/DB57/contig.2.DB57_length_702_mean_cov_6.59971509972.docx]

**DB57_length_702_mean_cov_6.59971509972**

```
ACCATGTGAGGTGAATGATGGCATATGGAAATGTGTACTAATATAAAATGGGCTTTGTGGTTAATCAATAATGCGTTAATAGATTATGG
 >chr7:126433918-126434278 + E=9e-187
GAAATAATGACACCAGTAAGGAATTACTCTGGATGATTAATAGCAGTAGAGATTTCCATGAATTTAAATGGCCCTTGAAAATAACACAT

AATATTTACTTGCCTGGGTTTTCTTTAAGAAAAATATCGATTTGAAACTCAGAAATCACAAAAACAAAGAGGCTAAAATCAGACAGAAT

GTCTTTGCTCTATTACTGAAACCACATAAAAAATAAAATAAAAAAAAAAGCCCTTAGCACTAGCAGTGTCAATTATCCACATTATTTAT

CCTG|GACA|TGGAAACTCTCATTTTATTTAGTATTTTCTAAGTATTTCCTAAAAATGATGTTTTATTTTAAAAAGATGAGTCTTATGT
         >chr7:126435203-126435541 - E=1e-188
TGATACTTTTCATTTATTATAAACTTACCTAAAAATCAGTCAACACCTGTAAAATTTTGGGGAAAACAAATGAGTATATGAGATAGTGG

AGCTGTGATGGGTACTTCACTTTGCTTCTGAGCTAGATTGTTTCAACTCTGCGCCTAATGCCTGAGAGTTTGTGTATAGTCATATCTTT

CGATATCTTTAGAGCCTAGGTTTAGTATCAGTTGTTTTCAATCTCAACTGTCCAGTTGACTCAAAAAGTAAAGCTTTTTAA
```
